# Letter to the Editor: The Implications of an Ab Interno Versus Ab Externo Surgical Approach on Outflow Resistance of a Subconjunctival Drainage Device for Intraocular Pressure Control

**DOI:** 10.1167/tvst.9.10.17

**Published:** 2020-09-15

**Authors:** Laszlo Romoda

**Affiliations:** Product Development, Allergan, an AbbVie company, Irvine, California. e-mail: laszlo.romoda@allergan.com

We read with interest the article titled “The Implications of an Ab Interno Versus Ab Externo Surgical Approach on Outflow Resistance of a Subconjunctival Drainage Device for Intraocular Pressure Control” by Lee et al.,^1^ the subsequent critical response to the article by Sheybani, Grover, and Fellman^2^ and the reply from the authors of the article.^3^

Allergan R&D would like to correct the assertion in the response from the authors that the origin of the pressure drop variability between the findings of Sheybani et al. and Lee et al. are due to manufacturing variability of the internal diameter of the XEN implant, as stated by Lee et al.

XEN products are designed to provide safe, effective pressure drop to treat glaucoma. The manufacturing process yields far tighter control over pressure drop than the assertion implies. Furthermore, the XEN numerical product designation is not meant to be a product specification nor indication of manufacturing tolerance. Therefore, calculating expected pressure drop from the product name will not result in a meaningful value.

Additionally, we would greatly appreciate it if you could ensure that this explanation travel with the manuscript as is customary.
